# UCHL3 promotes hepatocellular carcinoma progression by stabilizing EEF1A1 through deubiquitination

**DOI:** 10.1186/s13062-024-00495-w

**Published:** 2024-07-04

**Authors:** Jie Zhao, Qiang Huo, Ji Zhang, Kexiang Sun, Jinhui Guo, Feng Cheng, Xiaoge Hu, Qiuran Xu

**Affiliations:** 1 College of Biotechnology and Bioengineering, Zhejiang University of Technology, Hangzhou, China; 2 Zhejiang Key Laboratory of Tumor Molecular Diagnosis and Individualized Medicine, Zhejiang Provincial People’s Hospital, Affiliated People’s Hospital, Zhejiang Provincial People’s Hospital, Hangzhou Medical College, Hangzhou, China; 3 Department of General Surgery, Zhoushan Dinghai Central Hospital (Dinghai District of Zhejiang Provincial People’s Hospital), Zhoushan, China; 4 Qingdao Medical College, Qingdao University, Qingdao, China

**Keywords:** Hepatocellular carcinoma, EEF1A1, UCHL3, Deubiquitination, Stemness

## Abstract

**Background:**

Hepatocellular carcinoma (HCC) ranks as the second leading cause of global cancer-related deaths and is characterized by a poor prognosis. Eukaryotic translation elongation factor 1 alpha 1 (EEF1A1) have been proved to play important roles in various human cancers, whereas the deubiquitination of EEF1A1 was poorly understood.

**Methods:**

The binding and regulatory relationship between Ubiquitin carboxyl-terminal hydrolase L3 (UCHL3) and EEF1A1 was validated using clinical tissue samples, reverse transcription quantitative real-time fluorescence quantitative PCR (RT-qPCR), Western blotting, co-immunoprecipitation, and immunofluorescence, as well as ubiquitin detection and cyclohexamide tracking experiments. Finally, the impact of the UCHL3/EEF1A1 axis on HCC malignant behavior was analyzed through functional experiments and nude mouse models.

**Results:**

UCHL3 was found to have a high expression level in HCC tissues. Tissue samples from 60 HCC patients were used to evaluate the correlation between UCHL3 and EEF1A1. UCHL3 binds to EEF1A1 through the lysine site, which reduces the ubiquitination level of EEF1A1. Functional experiments and nude mouse models have demonstrated that the UCHL3/EEF1A1 axis promotes the migration, stemness, and drug resistance of HCC cells. Reducing the expression of EEF1A1 can reverse the effect of UCHL3 on the malignant behavior of HCC cells.

**Conclusion:**

Our findings revealed that UCHL3 binds and stabilizes EEF1A1 through deubiquitination. UCHL3 and EEF1A1 formed a functional axis in facilitating the malignant progression of HCC, proving new insights for the anti-tumor targeted therapy for HCC.

**Supplementary Information:**

The online version contains supplementary material available at 10.1186/s13062-024-00495-w.

## Introduction

Hepatocellular carcinoma (HCC) ranks among the top ten global malignant tumors, presenting challenges in early diagnosis and exhibiting a high recurrence rate [1, 2]. Surgical interventions, such as liver resection or transplantation, represent significant treatment modalities for patients in the early or intermediate stages of the disease [3]. However, for those in advanced stages, surgical approaches are prone to relapse and deterioration, the efficacy of drugs and other therapies remains unsatisfactory. Therefore, a comprehensive understanding of the molecular mechanisms underlying the onset and progression of HCC is crucial for identifying novel therapeutic targets that could enhance overall survival for patients with HCC.

Ubiquitination, a potent post-translational modification, plays a pivotal role in regulating various intracellular physiological processes, including cell growth, differentiation, transcriptional regulation, and carcinogenesis [4]. This process is reversible, and deubiquitinating enzymes (DUBs) play a crucial role in removing ubiquitin from substrates. Numerous studies have highlighted the close association of DUBs with human cancer development, positioning them as potential targets for tumor therapy [5]. Ubiquitin C-Terminal Hydrolase L3 (UCHL3) is a member of the ubiquitin c-terminal hydrolases (UCH) family within DUBs and several proteins including FOXM1, LDHA, AhR, TRAF2 and Vimentin have been revealed to be the substrates of UCHL3 by acting as a DUB. UCHL3 enhances drug resistance and glycolysis in pancreatic cancer through the stabilization of FOXM1 and upregulation of LDHA [6, 7]. In lung adenocarcinoma, UCHL3 stabilizes the aryl hydrocarbon receptor (AhR), contributing to the acquisition of stemness features [8]. In ovarian cancer, UCHL3 activates NF-κB signaling by deubiquitinating and stabilizing TRAF2 [9]. Moreover, UCHL3 regulates sex-determining region Y-box 12 (SOX12) through the AKT/mTOR signaling pathway, promoting the proliferation of colorectal cancer cells [10]. Despite these findings, our previous research found a regulatory relationship between UCHL3 and vimentin in HCC. Other substrates of UCHL3 needs to be further investigated.

Eukaryotic translation elongation factor 1 alpha 1 (EEF1A1), a member of the EEF1A family, has been implicated in promoting the progression of various cancers, including lung cancer, colorectal cancer, breast cancer, gastric cancer, ovarian cancer, etc. [11–15]. In HCC cells, EEF1A1 exhibits high expression and is involved in the regulation of cell cycle, invasion, metastasis, proliferation, and other unfavorable behaviors of HCC cells [16–19]. Earlier investigations have indicated that the ubiquitin-like protein FAT10 is capable of stabilizing the expression of EEF1A1. Nonetheless, it is yet to be elucidated whether UCHL3 is involved in the deubiquitination of EEF1A1.

In this study, we revealed EEF1A1 was a binding protein of UCHL3 according to the mass spectrometry (MS) analysis of co-immunoprecipitation complex with UCHL3 antibody. UCHL3, acting as a DUB, stabilized the protein level of EEF1A1, therefore enhancing the stemness and migration of HCC cells. Notably, depleting EEF1A1 could reverse the impact of UCHL3 on the malignant behaviors of HCC cells and reduce the drug resistance of HCC to Lenvatinib. Additionally, we further explored the precise binding sites of EEF1A1 with UCHL3, and their binding was necessary for the function of UHCL3 in HCC. Altogether, our findings underscore the potential of the UCHL3/EEF1A1 axis as a novel therapeutic strategy for HCC.

## Materials and methods

### Cell culture

HCC cell lines (HUH7, HEP3B) used were purchased from the Chinese Academy of Sciences cell library (https://www.cellbank.org.cn/index.php). HUH7 cells were cultured using DMEM (VivaCell, C3060-0500) medium containing 10% fetal bovine serum (BI, 04001–1 A) and 1% penicillin-streptomycin (Cienry, CR15140). HEP3B cells were cultured using MEM (VivaCell, C3060-0500) medium containing 10% fetal bovine serum (BI, 04001–1 A) and 1% penicillin-streptomycin (Cienry, CR15140). The cells were grown in an incubator containing 5% CO_2_ at 37℃.

### Virus infection and siRNA transfection

The lentivirus with UCHL3 overexpression specificity was purchased from GeneChem. The lentivirus for UCHL3 knockdown was purchased from Genomeditech. The siRNA targeting EEF1A1 was purchased from Shanghai GenePharma. The cells were seeded into 6-well plates. After 24 h, the lentivirus was dissolved in the medium and added to each well. After overnight incubation, the supernatant was replaced with fresh medium, and the culture was continued for 48 h. The shRNAs sequences of the genes are in Supplementary Table S1.

### Wound healing assay

A certain amount of HCC cells was collected and inoculated on both sides of the scratch chamber. Medium containing 10% fetal bovine serum and 1% penicillin was used to culture the cells until they were attached to the wall. Then the scratch chamber was pulled out and the cells were cultured in the low-serum medium for culture. Finally, the healing rate of different groups of cells was compared at 0 h and 24 h respectively.

### Transwell migration assay

The chamber was placed in a 24-well culture plate. A certain amount of HCC cells was harvested and re-suspended in serum-free medium. 200 µL of cell suspension was absorbed and added to the upper compartment of each compartment, and 600 µL of medium containing 10% serum was filled in each lower compartment. Following incubation at 37 °C for 24 or 48 h, the chamber was gently washed twice with PBS, fixed with methanol, and stained using crystal violet staining solution.

### Co-immunoprecipitation and ubiquitination assay

The protein supernatant was obtained from HCC cell lines after lysis. Anti-UCHL3, anti-EEF1A1, anti-Flag and anti-UB antibodies were used to adsorb and bind with magnetic beads for 15 min. The supernatant was removed and the magnetic beads were cleaned three times. The cell lysate was added and rotated overnight in a shaker at 4℃. The magnetic beads were washed and boiled with 1×sample buffer for western blotting to observe the binding of proteins and the level of endogenous ubiquitination in cells. IgG was used as control throughout the experiment. In an in vitro deubiquitination experiment, the ubiquitin plasmid carrying the HA tag and EEF1A1 with the Flag tag were co-transfected into 293T cells, and MG132 was employed to inhibit proteasomal degradation, and then ubiquitinated EEF1A1 was purified by Flag antibody. Subsequently, the UCHL3 plasmid carrying the His tag was bacterially purified. Ubiquitin-labeled EEF1A1 was incubated with or without UCHL3 at 30 °C for 4 h in vitro, and the ubiquitination level of EEF1A1 was detected using the HA antibody tag.

### Western blot analysis

First, cell lysate sample were quantified using the BCA protein detection kit (Thermo Fisher, Cat#23225). The quantified protein samples were separated by sodium dodecyl sulfate-polyacrylamide gel electrophoresis (Yeasen, 20325ES62). The isolated protein samples were transferred to polyvinylidene fluoride (PVDF) membrane (Millipore, IPVH00010). The transferred membranes were enclosed in 5% skim milk (Yeasen, 36120ES76) at room temperature for 1 h and incubated overnight with the corresponding antibody working solution in a shaker at 4 °C. The information of the antibodies in the text is in Table S2. The next day, the membrane was cleaned three times with TBST (Solarbio, T1082), and then combined with anti-secondary rabbit or anti-secondary mouse antibodies were incubated at room temperature for about 1 h, and the membranes were cleaned three times again using TBST. Finally, the protein levels in the samples were detected by enhanced chemiluminescence solution (BIO-RAD).

### Spheroid formation assay

The stably transfected cells were collected and spread into low-adsorption 6-well plates. The cells were cultured in DMM-F12 (1:1) medium containing 10ng/ml EGF, 10ng/ml FGF, and N2 (ThermoFisher, Cat#17502048). A week later, the number and size of spheres were observed with a microscope, and the results were statistically analyzed.

### Reverse transcription quantitative real-time PCR (RT-qPCR)

Total RNA was extracted from different HCC cells using the Rapid RNA Extraction Kit (YiShan, RN001). The extracted RNA was reversely transcribed into cDNA using a quantitative kit (Takara, RR037A). RT-qPCR experiments were conducted using a reaction system containing forward and reverse primes and fluorescent dye SYBR Green (Yeasen, 11184ES08). Finally, 7500 system was used to measure the fluorescence intensity of SYBR Green. The sequence of primers is shown in supplementary Table S3.

### Site-directed mutation and PCR assay

The EEF1A1 gene sequence, incorporating the Flag tag, was procured from Sangon Biotech. Specific primers, incorporating mutation sites, were employed to precisely introduce mutations at designated lysine sites in EEF1A1. Subsequently, a PCR reaction was conducted to amplify the entire plasmid containing the EEF1A1 mutation site, followed by plasmid amplification in Escherichia coli (E. coli). The primer sequences required for the PCR reaction are detailed in Table S3 of the supplementary materials.

### Immunofluorescence colocalization

5 × 10^4^ cells were seeded in confocal dishes. After cell attachment, cells were fixed using formaldehyde. Afterwards, the formaldehyde-fixed cells were incubated overnight with Anti-UCHL3 (Proteintech, 12384-1-AP, 1:200) antibody and Anti-EEF1A1 (Proteintech, 67495-1-Ig, 1:200) antibody. The following day, the cells were exposed to fluorescently labeled secondary antibodies for one hour. After rinsing off the secondary antibody with Phosphate-Buffered Saline (PBS), anti-fade solution containing DAPI (Beyotime, P0131) was applied to the confocal dish. Consequently, the treated cells were observed under a fluorescence microscope.

### Construction of drug-resistant cells

The half-maximal drug inhibitory concentration (IC50) of wild-type HUH7 and HEP3B cells for Lenvatinib was evaluated. HCC cells were treated with Lenvatinib, and Lenvatinib-resistant cell lines were developed during the culture process.

### Cell counting kit-8 (CCK8)

The cells were uniformly distributed across a 96-well plate, cultured until they adhered to the wall, and then treated with the corresponding drug for 3–4 days. The culture medium was mixed with the CCK8 solution in a 10:1 ratio and added to the 96-well plate in the absence of light. After incubation at 37 °C for 2 h, the absorbance (OD) value of each well at 450 nm was measured using an reader. Based on the results, the IC50 value of Lenvatinib on the cells was calculated.

### Proteomic analysis

Firstly, collect HUH7 cell lines overexpressing UCHL3 and extract the total protein. Immunoprecipitation was performed using UCHL3 antibodies, with IgG serving as the negative control. Weastern blotting was employed to separate the protein samples from the immunoprecipitation, and the gel strips of all protein samples were cut and subsequently subjected to enzymatic hydrolysis. Subsequently, liquid chromatography-mass spectrometry (LC-MS) was utilized to analyze the enzymatic hydrolysis products of rubber strips. The mass spectrometry results were then compared with the gene database to identify the product genes.

### Animals

Female mice aged 4-5 weeks were procured from Shanghai Laboratory Animal Science and Technology Company. Subcutaneous tumor formation in nude mice was performed using a HUH7 cell line stably transfected with UCHL3. The weight and tumor size of nude mice were measured at weekly intervals. After 3-4 weeks, the nude mice were anesthetized and killed, and the subcutaneous tumors of the nude mice were removed and the size and volume of the tumors were measured. All above animal procedures are carried out in accordance with the ethics approved by Zhejiang Provincial People’s Hospital.

### Statistical analysis

All experimental data were analyzed and calculated by statistical software program (Graphpad, 8.0, San Diego, CA, USA) and ImageJ. All data were collected from three independent experiments. Single-factor analysis of variance is employed to discern disparities among multiple groups. The disparities between two groups in the article were assessed by t-test. ANOVA test was used when there were three or more groups. P-values less than 0.05 were generally considered statistically significant. (*p < 0.05, **p < 0.01, ***p < 0.001).

## Results

### UCHL3 emerges as an unfavorable prognostic biomarker for HCC

Our findings indicated a potential association between UCHL3 expression in HCC and the age and gender of patients (Figure S1A-B). The Human Protein Atlas reveals a significant elevation of UCHL3 in HCC tissue compared to adjacent HCC tissues (Figure S1C). Furthermore, a strong correlation between UCHL3 and the stage of HCC patients was also observed (Figure S1D, Table 1).

**Table 1:** Correlation between UCHL3 expression and clinicopathological features of HCC

Characteristics		*n* = 60	UCHL3 expression		*P* value
			Negative (*n* = 23)	Positive (*n* = 37)	
Age (years)	< 50	26	11	15	0.5798
	≥ 50	34	12	22	
Sex	Male	47	17	30	0.5123
	Female	13	6	7	
HBV infection	No	21	11	10	0.1005
	Yes	39	12	27	
Serum AFP level (ng/mL)	< 20	20	10	10	0.1887
	≥ 20	40	13	27	
Tumor size (cm)	< 5	19	11	8	**0.0339**
	≥ 5	41	12	29	
No. of tumor nodules	1	49	20	29	0.4038
	≥ 2	11	3	8	
Cirrhosis	No	26	13	13	0.1041
	Yes	34	10	24	
Venous infiltration	No	33	16	17	0.0738
	Yes	27	7	20	
Edmondson-Steiner grade	I + II	43	20	23	**0.0382**
	III + IV	17	3	14	
TNM stage	I + II	46	21	25	**0.0346**
	III + IV	14	2	12	

UCHL3 negative: IHC score ≤ 3; UCHL3 positive: IHC score > 3

**Fig. 1:**
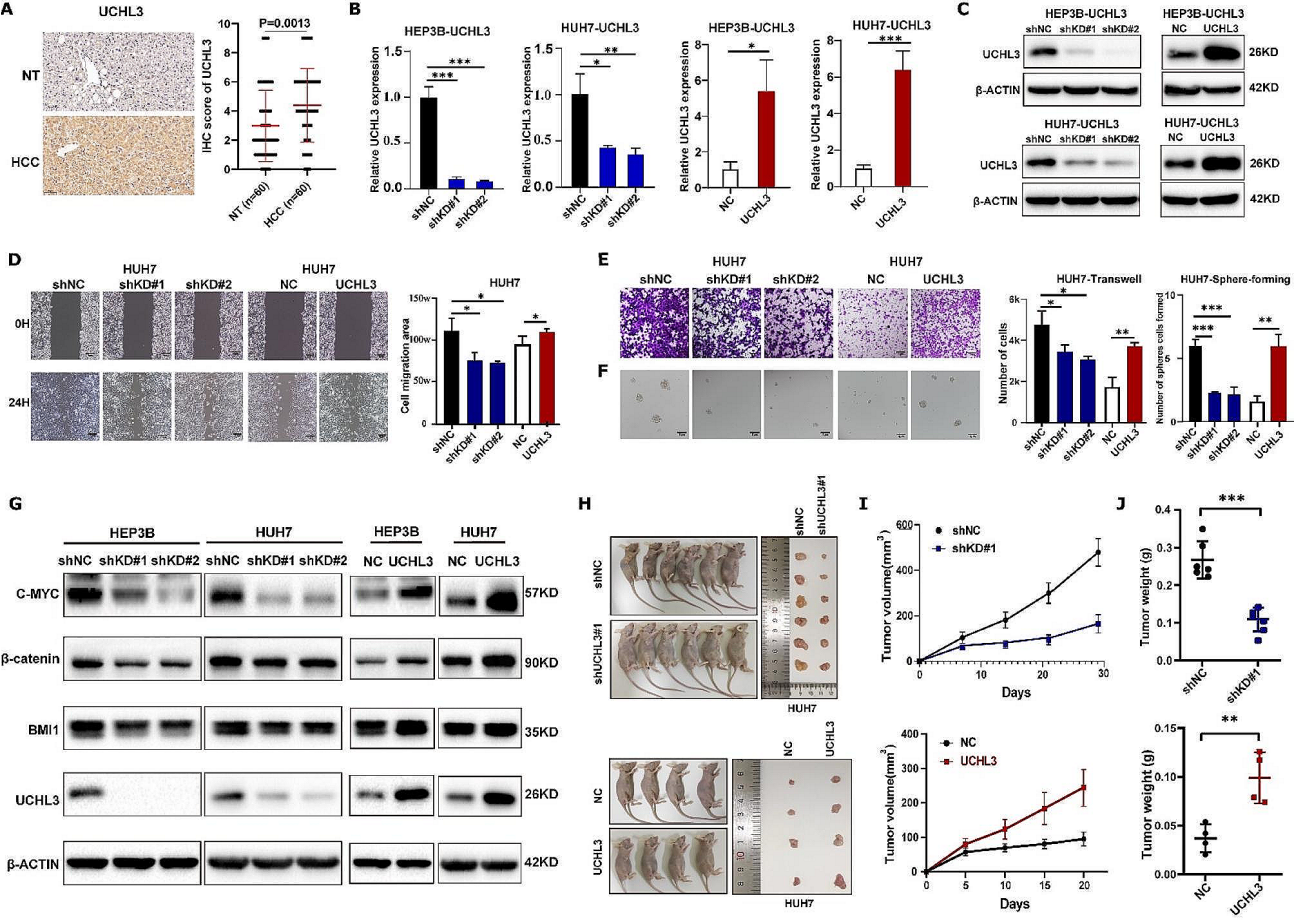
UCHL3 impacts the malignancy of HCC in vitro and in vivo. (**A**) IHC revealed a significant increase in the expression level of UCHL3 in HCC tissue compared to adjacent non-cancerous tissue. Data analysis showed statistical significance, *P* = 0.0013. (**B-C**) UCHL3 viral vectors were employed for transfection in two HCC cell lines (HEP3B, HUH7), and the transfection efficiency at the mRNA level was assessed via RT-qPCR (**B**). The transfection efficiency of intracellular protein levels of the two cell lines was detected by western blot (**C**). (**D**) The wound healing experiments indicated that UCHL3 significantly influences the migration area of the HCC cell lines. (**E**) Transwell assay was employed to evaluate the migratory potential of HCC cells following alterations in UCHL3 expression. (**F-G)** Sphere formation (**F**) and western blot experiments (**G**) further demonstrated that manipulating UCHL3 expression significantly influenced the stemness of HCC cells and the expression of stemness-related proteins. (**H**) Subcutaneous tumor image of UCHL3 knockdown and overexpression HUH7 cells. (**I-J**) Knockdown and overexpression of UCHL3 can influence tumor size (**I**) and tumor weight (**J**) as revealed by subcutaneous tumor mouse model

To validate the results, tumor tissues and adjacent normal tissues from 60 clinical HCC patients were collected. We observed an elevation in UCHL3 protein expression in HCC tissues (Fig. 1A). Next, we used lentivirus to construct UCHL3 stable knockdown and overexpression HCC cell lines (Fig. 1B-C). Following that, transwell and wound healing assay provided evidence that UCHL3 profoundly influences the migratory properties of HUH7 cells (Fig. 1D-E) and HEP3B cells (Figure S1E-F). Sphere formation assay showed that UCHL3 enhanced the sphere formation ability of cells (Fig. 1F, S1G). Western blot results also showed that UCHL3 promoted the expression levels of these stemness-related proteins (Fig. 1G). To validate the in vivo functionality of UCHL3, we conducted a subcutaneous tumor growth experiment using a nude mouse model (Fig. 1H). The observation of the growth volume and weight of tumors in nude mice indicates that UCHL3 significantly promoted the tumor growth of HCC cells (Fig. 1I-J). In summary, our experiments demonstrate that UCHL3 may be a potential therapeutic target for HCC.

### EEF1A1 is identified as a potential binding protein of UCHL3

**Fig. 2:**
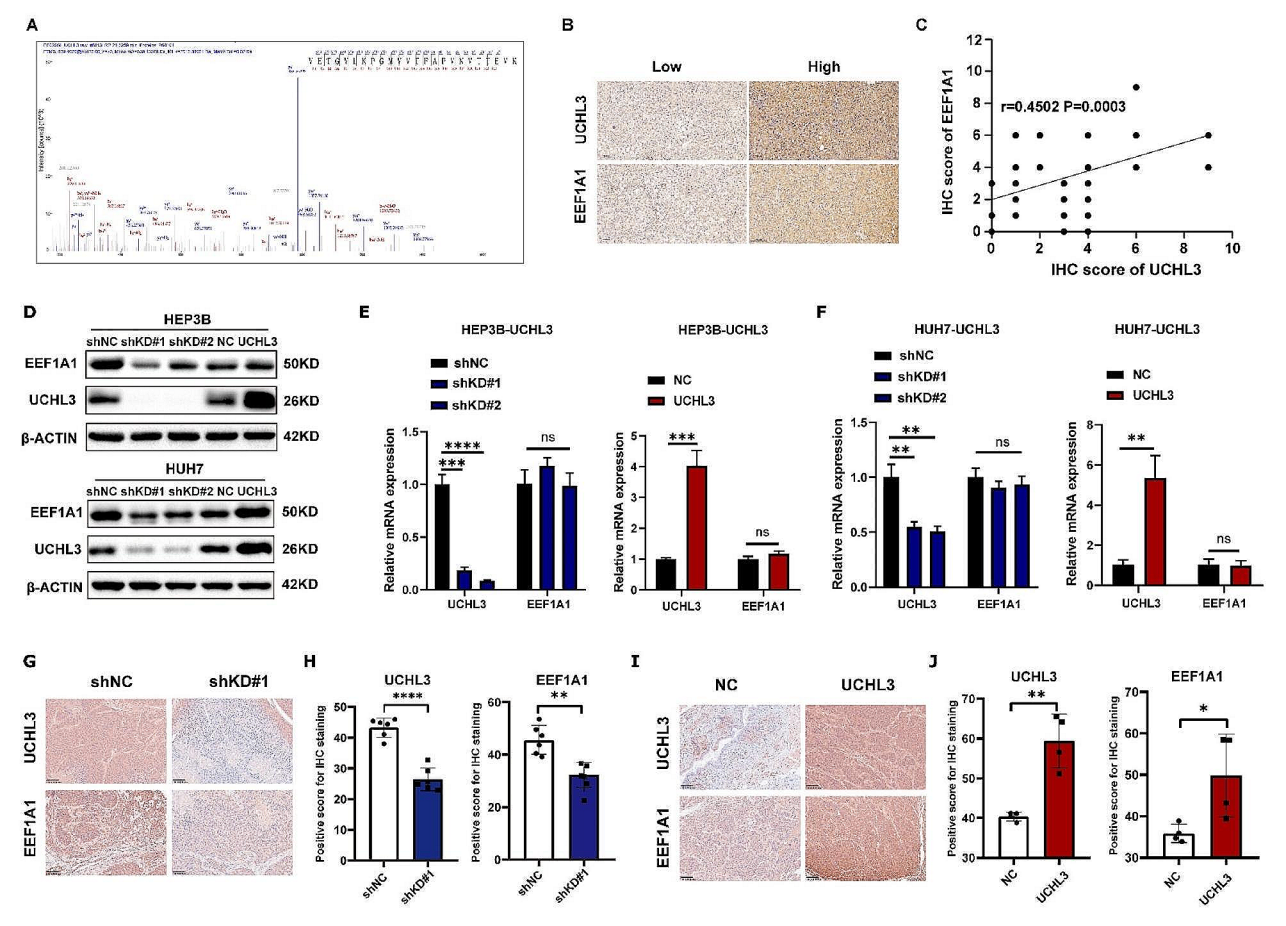
Identification the interaction between EEF1A1 and UHCL3. (**A**) In we detected the peptide of EEF1A1 in the mass spectrometry of immunoprecipitation complex of UCHL3. (**B**) Immunohistochemistry of a cohort of 60 HCC patients was conducted. Representative image of the expression of UCHL3 and EEF1A1 in human HCC tissues. (**C**) Statistical analysis (Simple linear regression) of the correlation between UCHL3 and EEF1A1 (*R* = 0.4502, *P* = 0.0003) in 60 HCC patients. (**D-F**) Protein (**D**) and RNA (**E-F**) expression of UCHL3 and EEF1A1 in UCHL3-overexpressed and UHCL3-knockdown HEP3B and HUH7 cells. (**G-H**) The modulation of EEF1A1 by UCHL3 was evaluated in the nude mouse tumors of UCHL3 knockdown group through immunohistochemistry (**G**). Statistical analysis indicated a decrease in EEF1A1 expression was found upon UCHL3 knockdown in mouse tumors (**H**). (**I-J**) Similarly, the expression of EEF1A1 and UCHL3 was analyzed by IHC in UCHL3 overexpression nude mouse tumors (**I**). Statistical analysis indicated an increase in EEF1A1 expression was observed upon UCHL3 overexpression in mouse tumors (**J**)

According to our previous immuniprecipitation and MS analysis with UCHL3 antibody, EEF1A1 was significantly enriched in UCHL3-immuniprecipitation group compared to IgG group (Fig. 2A). IHC was performed in human HCC tissues, and the representative image of the expression of UCHL3 and EEF1A1 were shown in Fig. 2B. Based on the statistical results of IHC score, there was a significant positive correlation between UCHL3 and EEF1A1 expression in HCC tissues (Fig. 2C). Next, we analyzed EEF1A1 expression after knockdown or overexpression of UCHL3. The results showed that UCHL3 depletion decreased EEF1A1 protein expression, whereas UCHL3 overexpression upregulated EEF1A1 expression (Fig. 2D). However, no change was observed in EEF1A1 mRNA levels, whether UCHL3 was overexpressed or knockdown (Fig. 2E-F). Thus, UCHL3 regulated the protein level of EEF1A1 rather than its mRNA level. IHC analysis of the tumor in nude mouse tumors showed that UCHL3 overexpression increased EEF1A1 protein level, while UCHL3 inhibition downregulated EEF1A1 protein level (Fig. 2G-J). In summary, EEF1A1 was regulated by and positively correlated with the protein level of UCHL3.

### EEF1A1 is bound and stabilized by the deubiquitinating action of UCHL3

**Fig. 3:**
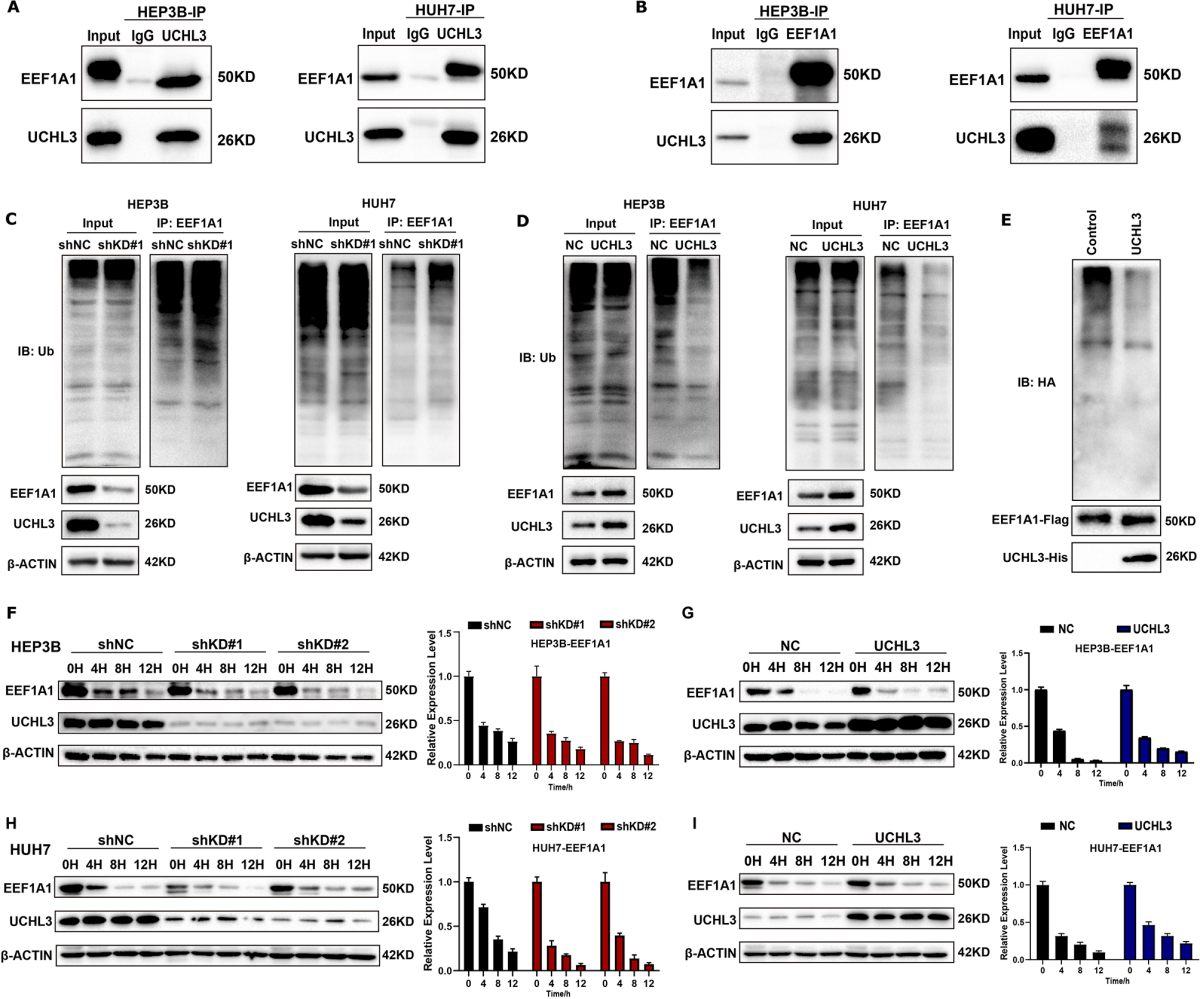
UCHL3 can bind to EEF1A1 and enhance the stability of EEF1A1 via ubiquitination. (**A-B**) HEP3B and HUH7 cells were used to detect the interaction between endogenous UCHL3 and EEF1A1 by immunoprecipitation. (**C-D**) UCHL3 can regulate the ubiquitination level of EEF1A1. After protein co-immunoprecipitation using anti-EEF1A1 antibody, anti-UB antibody was used to detect the ubiquitination level of EEFA1 due to the change of UCHL3 expression. (**E**) The expression of exogenous ubiquitinated EEF1A1 protein was purified from 293T cells, and the ubiquitination level of EEF1A1 was detected after incubation with or without UCHL3 in vitro. (**F-I**) UCHL3 knockdown (shNC, shKD#1, shKD#2) and UCHL3 overexpression (NC, UCHL3) of HEP3B cells were treated with cycloheximide (CHX, 10 µg/ ml) at 0 h, 4 h, 8 h, and 12 h, respectively(**F-G**). Similarly, the same experiment was carried out in HUH7 cells (**H-I**). The expression of EEF1A1 protein affected by UCHL3 was analyzed by western blot. The statistical result was shown on the right

To further investigate whether UCHL3 regulates EEF1A1 expression through deubiquitination. We verified the interaction between UHCL3 and EEF1A1 in HCC cells by co-immunoprecipitation (Fig. 3A-B). UCHL3 and EEF1A1 could bind with each other in both HEP3B and HUH7 cells. Next, we wonder whether UCHL3 regulated ubiquitination of EEF1A1. The results showed that the ubiquitination level of EEF1A1 was significantly increased upon loss of UCHL3 (Fig. 3C). On the contrary, overexpression of UCHL3 reduce the ubiquitination level of EEF1A1(Fig. 3D). In vitro deubiquitinaion assay further revealed the role of UCHL3 to deubiquitinate EEF1A1 (Fig. 3E). Subsequently, we investigated the half-life of EEF1A1 protein in HCC cell lines after treatment with cycloheximide (CHX). Knockdown of UCHL3 could promote the degradation of EEF1A1, and overexpression of UCHL3 increased the stability of EEF1A1 (Fig. 3F-I). In conclusion, EEF1A1 was novel substrate protein of UCHL3, and UCHL3 can bind to and then stabilize EEF1A1 protein through removing ubiquitin modifications from EEF1A1.

### EEF1A1 binds to UCHL3 through the lysine site

**Fig. 4:**
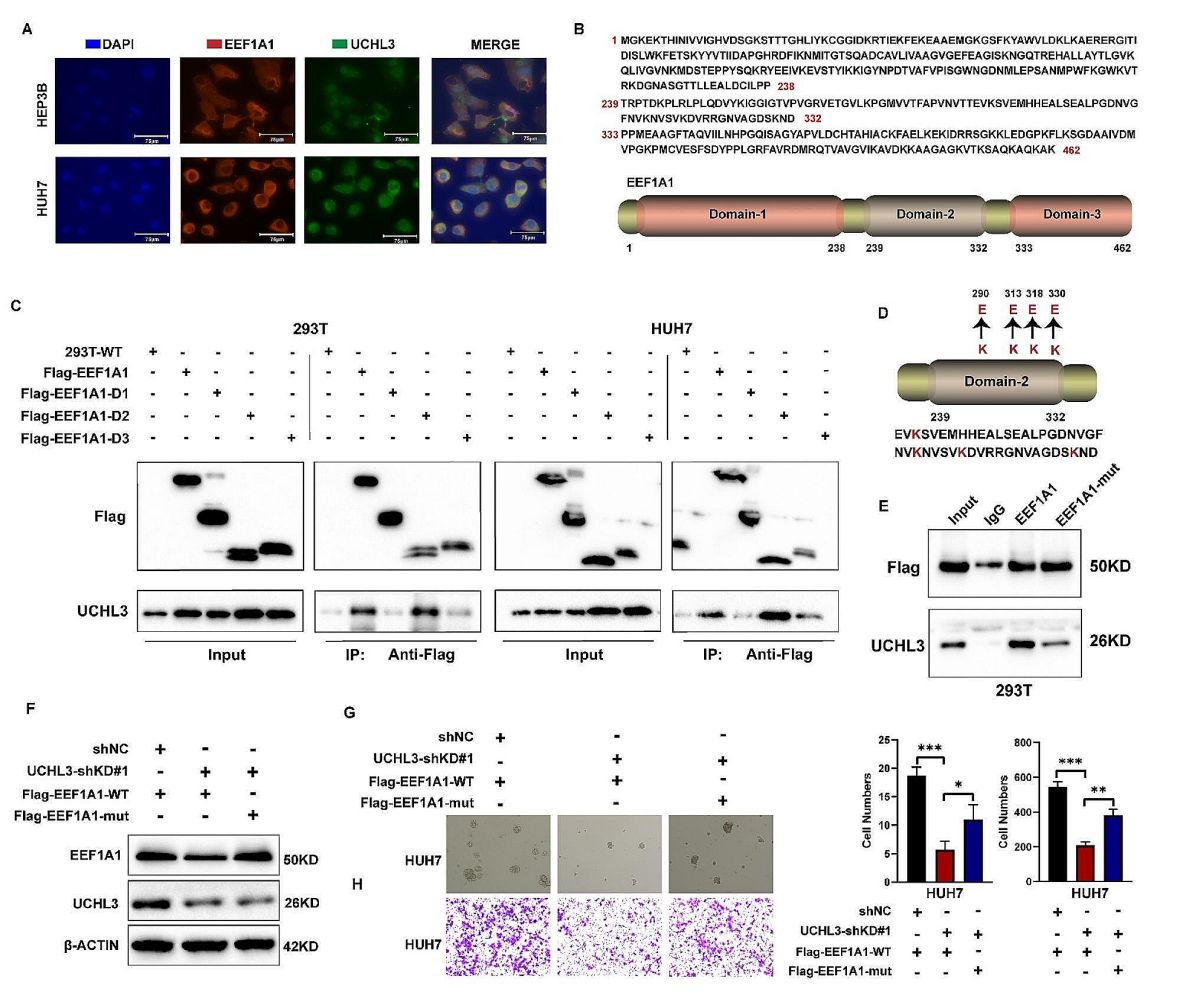
The domain 2 was important for EEF1A1 to interact with UCHL3. (**A**) Immunofluorescence staining and confocal microscopy were used to detect the binding of EEF1A1 and UCHL3 in HEP3B and HUH7 cells. (**B**) The protein sequence of EEF1A1, as well as the nucleotide sequence and schematic diagram of the three truncated proteins. (**C**) Immunoprecipitation analysis of UCHL3 binding regions on EEF1A1 in 293T cells and HUH7 cells. (**D**) Mutate the lysine at position 290, 313, 318, 330 of domain 2 in EEF1A1 to arginine. (**E**) Immunoprecipitation analysis of the effect of lysine mutation of EEF1A1 on the interaction between UCHL3 and EEF1A1 in 293T cells. (**F**) The western blot results indicated that UCHL3 does not modulate the protein expression level of EEF1A1-mut in HUH7 cells. (**G-H**) Sphere formation assay and transwell experiments illustrated that UCHL3 modulated the malignant phenotype of HCC by interacting with EEF1A1 in HUH7 cells

Our above findings showed that UCHL3 could bind to EEF1A1 and mediated its deubiquitination. Therefore, we aimed to elucidate the underlying molecular mechanism of UCHL3 binding to EEF1A1. First, we verified the interaction between UHCL3 and EEF1A1 in HCC cells by immunofluorescence (Fig. 4A). Next, we construct the Flag-tagged truncated plasmids of EEF1A1 containing domain 1, domain 2 or domain 3 separately (Fig. 4B). In order to identify the specific binding regions of EEF1A1 and UCHL3, we transfected Flag-tagged full-length EEF1A1 or its truncated proteins in 293T cells and HUH7 cells followed by immunoprecipitation with Flag antibody. The results showed that the domain 2 (aa239 to aa332) of EEF1A1 mainly mediated its interaction with UCHL3 (Fig. 4C). Previous research has indicated that EEF1A1 primarily interacts with UB molecules through lysine residues within the aa290 to aa332 range [20]. To precisely identify the lysine binding site of UCHL3 on EEF1A1, we performed sequential mutations on EEF1A1, changing lysine to arginine at four specific sites within the aa290 to aa332 of EEF1A1 (Fig. 4D). The results of protein co-immunoprecipitation revealed a reduced binding between EEF1A1 and UCHL3 after four lysine sites are mutated, indicating that UCHL3 primarily interacts with EEF1A1 through these sites (Fig. 4E). To further illustrated the effect of the binding between EEF1A1 and UCHL3 on the function of UCHL3, we conducted transwell and sphere formation assays. We transfected wide-type or mutated EEF1A1 to HCC cells (Fig. 4F). The experimental results showed that UCHL3 knockdown reduced the protein level of EEF1A1, and the migration ability and sphere formation ability were decreased, which was alleviated in mutated EEF1A1 group (Fig. 4G-H). Overall, our above findings elucidated that UCHL3 predominantly interacted with the lysine site within the second domain of EEF1A1.

### The EEF1A1/UCHL3 axis promotes HCC cell malignant and drug resistance

**Fig. 5:**
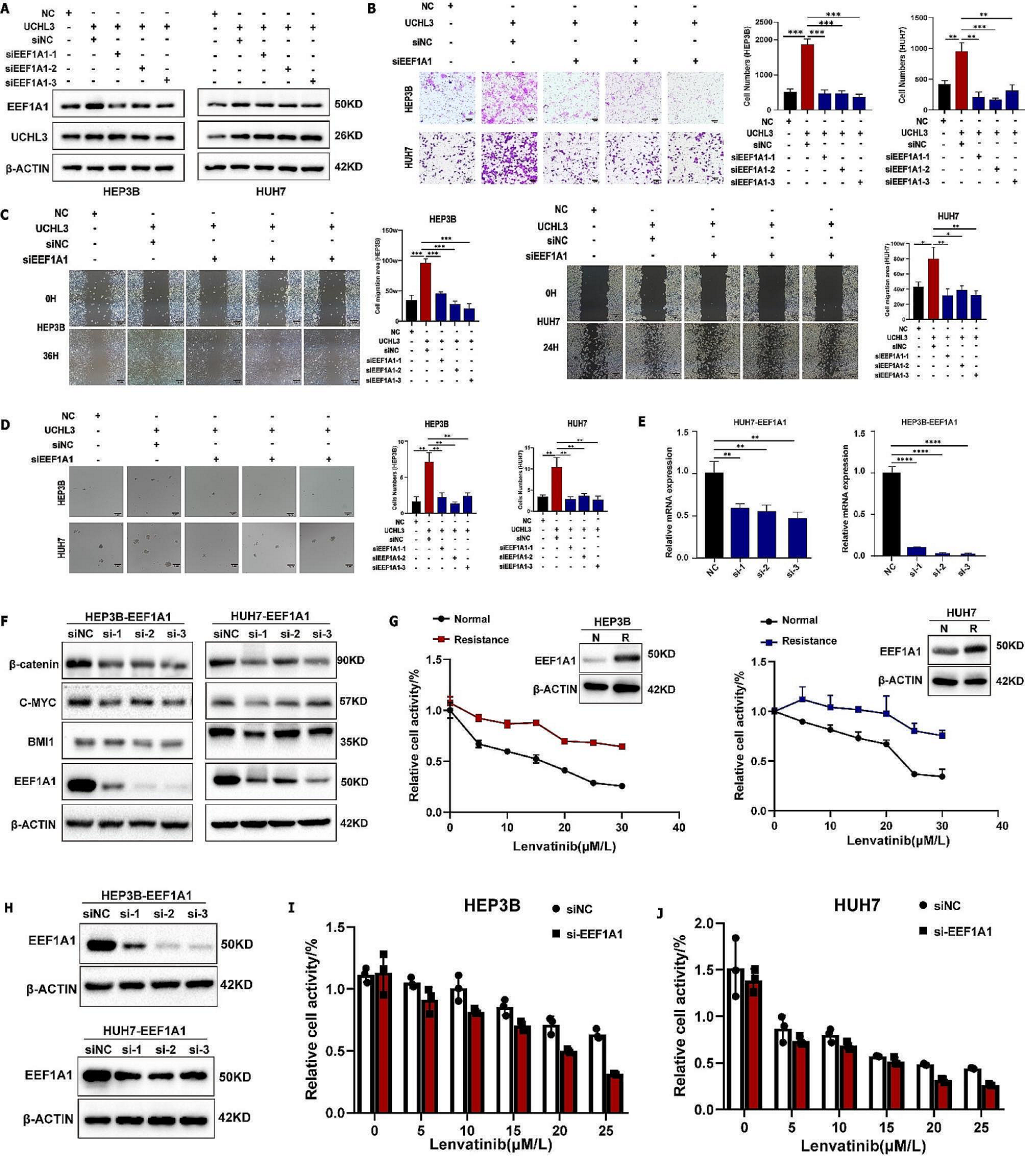
The oncogenic function of UCHL3/EEF1A1 axis in HCC cells. (**A**) western blot analysis of knockdown EEF1A1 in UCHL3 overexpression HEP3B and HUH7 cells. (**B**) Transwell assay indicated that EEF1A1 inhibition suppressed the enhanced migration ability of UCHL3. (**C**) Wound healing assay showed that reducing the expression of EEF1A1 can attenuate the promotive impact of UCHL3 on the migration function of HEP3B and HUH7 cells. (**D**) EEF1A1 knockdown reduced the increase of sphere formation ability of HEP3B and HUH7 cells. (**E**) HUH7 and HEP3B cells were transfected with the siRNA of EEF1A1, and the mRNA level transfection efficiency was detected by RT-qPCR. (**F**) Western blot was used to detect the interference efficiency of HUH7 and HEP3B cells on EEF1A1 at the protein level and the effect on the expression of related stemness proteins. (**G**) Expression of EEF1A1 protein in drug-resistant cell lines HEP3B and HUH7. (**H**) Transfection of EEF1A1 siRNA plasmids into two strains of cells resistant to Lenvatinib. (**I-J**) CCK8 assay was used to evaluate the effect of knockdown of EEF1A1 (si-2) on drug resistance of two HCC cell lines

Research suggests a strong association between EEF1A1 and poor prognosis in hepatocellular carcinoma, indicating its role as a pathogenic gene in this disease [16]. The functional rescue experiments were conducted to explore whether UCHL3 affected the malignant behavior of HCC by regulating EEF1A1. Firstly, siRNA was used to knockdown the protein expression of EEF1A1 in UCHL3 overexpressing HCC cells, and the knockdown level was detected by western blot (Fig. 5A). Transwell assay suggested that knockdown of EEF1A1 significantly attenuated the HCC cells migration enhancement induced by UCHL3 overexpression (Fig. 5B). In the wound healing assay, we also obtained the same conclusion (Fig. 5C). In addition, the depletion of EEF1A1 was observed to attenuate the spheroid-forming ability induced by UCHL3 overexpression in HCC cells (Fig. 5D). According to previous studies, EEF1A1 is associated with drug resistance in prostate cancer [21]. Therefore, we investigated whether EEF1A1 can induce the stemness characteristics of HCC and trigger the resistance mechanism of HCC. RT-qPCR and western blot demonstrated that knocking down the expression of EEF1A1 in HCC cells significantly reduces the expression levels of stemness proteins (Fig. 5E-F). Two HCC cell lines resistant to Lenvatinib were successfully constructed. The protein level of EEF1A1 was found to be upregulated in drug-resistant cells, indicating that EEF1A1 may be a potential resistance gene for Lenvatinib (Fig. 5G). To further validate the resistance mechanism of EEF1A1 in HCC, we used siRNA to reduce the expression level of EEF1A1 in drug-resistant cells (Fig. 5H). We observed that the absence of EEF1A1 (si-2) enhances the susceptibility of drug-resistant cells to Lenvatinib (Fig. 5I-J). Taken all the above findings together, The high expression of EEF1A1 can induce the stemness characteristics of HCC cells and trigger the resistance of HCC cells to Lenvatinib.

## Discussion

The UCH family has been extensively associated with human tumors in prior investigations [8, 22–24]. UCHL3 and UCH37 are acknowledged oncogenes, while BAP1 is recognized as a tumor suppressor. Previous reports suggest that UCHL3 is closely linked to signaling pathways such as NF-κB, AKT, TGF-β, and others in tumorigenesis [10]. UCHL3 enhances cancer cell stemness through intermediary receptor molecules like AhR [8]. It can enhance the radiosensitivity of non-small cell lung cancer cells by inhibiting DNA repair [25]. UCHL3 promotes the malignant progression of esophageal carcinoma by influencing CRY2 methylation [26]. Nevertheless, the precise mechanism of UCHL3 in HCC remains incompletely understood. In a prior study, we discovered that UCHL3 regulates the substrate molecule vimentin through ubiquitination in HCC [27]. However, the extent of UCHL3’s influence on EEF1A1 has not yet been verified. MS results indicate that the deubiquitination activity of UCHL3 can regulate multiple pathway targets, possibly involving more intricate molecular mechanisms.

In previous reports, EEF1A1 has been associated with a poor prognosis in colorectal cancer and HCC [28, 29]. It can affect the proliferation, apoptosis, invasion and metastasis of gastric cancer cells [14, 30]. However, some studies suggest that EEF1A1 does not influence cell apoptosis, possibly due to differences in cancer types. EEF1A1 also affects the development of tumors by promoting METTL13 methylation [31]. In addition, EEF1A1 has been reported to be regulated by the ubiquitination of FAT10 [20]. Here, we identify the EEF1A1 as downstream binding protein of UCHL3 using IP-MS.

Currently, we first identified EEF1A1 as a novel substrate protein of UCHL3 through its deubiquitination activity. UCHL3 upregulates the expression of EEF1A1 via deubiquitination, facilitating the migration of HCC cells through lysine-based interactions. The accumulation of EEF1A1 confers resistance to Lenvatinib in HCC cells, enhancing their stem-like properties. Additionally, UCHL3 and EEF1A1 enhance the growth capacity of subcutaneous tumors in nude mice. This indicates that the UCHL3 and EEF1A1 axis facilitate HCC cell proliferation in animal models. However, the effects of the UCHL3/EEF1A1 axis on HCC tumors as revealed by patient-derived xenograft (PDX) models remains to be explored.

Therefore, UCHL3 and EEF1A1 jointly promote the migration, stemness, and drug resistance of HCC cells, as well as influencing the growth of mouse tumor models. This suggests that UCHL3 may influence the malignant behavior of HCC by targeting EEF1A1. The present study did not further investigate the impact of the UCHL3/EEF1A1 axis on malignant behavior in HCC patients, thus presenting certain limitations. Further research can validate and develop targeted drugs against the UCHL3/EEF1A1 axis. Consequently, our research offers novel insights and ideas for the development of targeted clinical therapies for HCC.

### Electronic supplementary material

Below is the link to the electronic supplementary material.

Supplementary Material 1

## Data Availability

No datasets were generated or analysed during the current study.
