# Supplementary material for: UCHL3 promotes hepatocellular carcinoma progression by stabilizing EEF1A1 through deubiquitination
Source: Biol Direct. 2024 Jul 4;19:53. doi: 10.1186/s13062-024-00495-w (PMC11225194; doi:10.1186/s13062-024-00495-w)
Supplement: Supplementary file 1 — Supplementary Material 1 [file 13062_2024_495_MOESM1_ESM.docx]

**Supplementary material-Figure 1**

**
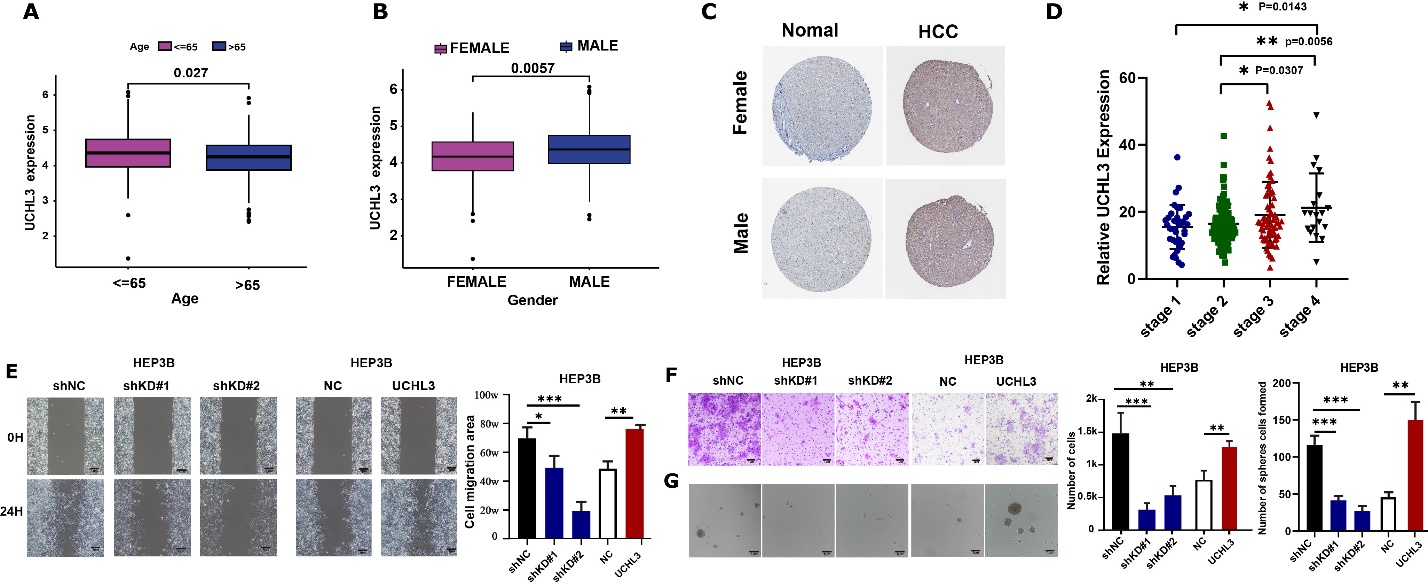
**

**Supplementary material-Tables**

**Table S1:** **All relevant sequences of transfected genes.**

| **Gene name** | **sequence** |
| --- | --- |
| UCHL3-shNC | 5'-TTCTCCGAACGTGTCACGT-3' |
| UCHL3-shKD1 | 5'-GGTCAGACTGAGGCACCAAGT-3' |
| UCHL3-shKD2 | 5'-GGAGGAATCTGTGTCAATGAG-3' |
| UCHL3-OENC | 5'-ATGAGCCCTGAAGAACGAGC-3' |
| UCHL3-OE | 5'-TCTGACCTTCATGGGCACTG-3' |
| EEF1A1-NC | 5'-UUCUCCGAACGUACGUTT-3' |
|  | 5'-ACGUGACACGUUCGGAGAATT-3' |
| EEF1A1-siRNA1 | 5'-GGCCAUCUGAUCUAUAAAUTT-3' |
|  | 5'-AUUUAUAGAUCAGAUGGCCTT-3' |
| EEF1A1-siRNA2 | 5'-GGCCCUAAAUUCUUGAAGUTT-3' |
|  | 5'-ACUUCAAGAAUUUAGGGCCTT-3' |
| EEF1A1-siRNA3 | 5'-CCAGGACACAGAGACUUUATT-3' |
|  | 5'-UAAAGUCUCUGUGUCCUGGTT-3' |

**Table S2:** **The antibody information used in the article.**

| **Gene name** | **Brand and Cat No** | **dilution ratio** |
| --- | --- | --- |
| UCHL3 | 12384-1-AP, Proteintech | 1:1000 |
| EEF1A1 | 67495-1-Ig, Proteintech | 1:1000 |
| β-ACTIN | AA128, Beyotime | 1:1000 |
| c-Myc | ab32072, Abcam | 1:1000 |
| β-catenin | 11834-3-AP, Proteintech | 1:1000 |
| Anti- Ubiquitin | P4D1, Santa | 1:1500 |
| Anti-Flag | 80010-1-RR, Proteintech | 1:3000 |

**Table S3:** **The primer sequences used in the article.**

| **Gene name** | | **Primer sequence** |
| --- | --- | --- |
| UCHL3 | Forward | 5'-CTGAAGAACGAGCCAGATAC-3' |
|  | Reverse | 5'-GCCCATCTACATGAACTAATGC-3' |
| β-ACTIN | Forward | 5'-TGA CCC AGA TCA TGT TTG AG-3' |
|  | Reverse | 5'-CGT ACA GGG ATA GCA CAG-3' |
| EEF1A1 | Forward | 5'-TGTCGTCATTGGACACGTAGA-3' |
|  | Reverse | 5'-ACGCTCAGCTTTCAGTTTATCC-3' |
| EEF1A1-mut1 | Forward | 5'-ACCGAGGTGCGTAGCGTGGA-3' |
|  | Reverse | 5'-ACGCACCTCGGTTGTCACGTTCAC-3' |
| EEF1A1-mut2  EEF1A1-mut3 | Forward | 5'-GCTTTAACGTGAGAAACGTGAGCGTGAGAGATGTGA-3' |
|  | Reverse | 5'-TCTCACGTTAAAGCCCACGTTATCGCC-3' |
| EEF1A1-mut4 | Forward | 5'-GGGACTCCAGAAACGATCCCCCAAT-3' |
|  | Reverse | 5'-TTCTGGAGTCCCCGGCCACGTT-3' |
